# COVID-19: challenges and the impact on care in clinical settings in Cameroon

**DOI:** 10.11604/pamj.supp.2020.35.24929

**Published:** 2020-07-24

**Authors:** Etienne Ngeh Ngeh, Christopher Kuaban

**Affiliations:** 1 Research Organisation for Health Education and Rehabilitation-Cameroon (ROHER-CAM),; 2 Regional Hospital Bamenda, North West Region-Cameroon,; 3 Department of Physiotherapy, St. Louis University Douala-Cameroon

**Keywords:** COVID-19, challenges, clinical care, Cameroon

## Abstract

COVID-19 is a new disease of pandemic proportions. Since the announcement of the first confirmed case of COVID-19 in Cameroon early this year, there has been an increasing number of circulating videos and messages from families about the poor management of their loved ones in clinical care settings. This correspondence highlights the challenges posed by COVID-19 and its impact on clinical care of patients in Cameroon.

## Commentary

Since the announcement of the first confirmed case of COVID-19 in Cameroon early this year, there has been an increasing number of circulating videos and messages from families about the poor management of their loved ones in clinical care settings around the country. This has brought about untold suffering to both patients and their families. Patients leave their homes for the hospital generally under very uncomfortable circumstances. This can be due to an acute, sub-acute or chronic condition. When these patients approach a clinician or medical facility, they expect medical treatment and care given with all the knowledge and skill that will bring relief to their problem. The relationship takes the shape of a tacit contract based on good faith retaining the essential elements of tort [1]. A clinician and medical facility owe certain duties to their patient and a breach of any of these duties could be considered as negligence or malpractice on the part of the clinician or facility. This correspondence highlights the impact of the COVID-19 pandemic on clinical care of patients in Cameroon. COVID-19 has placed significant strain on our health care system given the fact that it is a new disease of pandemic proportions. The existing health system is faced with several challenges ranging from inability to provide effective prevention strategies to health care providers and the general population, as well as triaging and managing infected COVID-19 cases and providing adequate support and rehabilitation to survivors [2]. Cameroon like many other African countries with existing fragile health systems coupled with the lack of preparedness against major outbreaks like COVID-19 faces difficulties in rapidly mobilizing resources to contain the pandemic [3]. The lack of resources coupled with poor management further increases these challenges with diverse consequences.

Many health facilities providing clinical care in Cameroon are challenged with insufficient availability of basic protective materials for health personnel and the human resource capacity to adequately tackle COVID-19. The lack of essential personal protective equipment such as the N95 or surgical facemasks, fluid resistant long-sleeve gowns, goggles recommended for clinicians are rarely available even in referral hospitals. The lack of specialist clinicians, technological and infrastructural resources to support health care providers during this period especially on issues of COVID-19 is challenging. Many health facilities lack the proper management systems to aid clinicians conduct proper triage and manage suspected cases effectively. This is further compounded by the lack of willingness and or potential to provide adequate trainings to aid in the process. Also, many health facilities in Cameroon are faced with serious challenges of setting up conducive pre-isolation and isolation centres making quarantine of suspected and confirmed cases respectively difficult with very poor compliance and poor outcomes for severe cases. It is generally accepted in clinical medicine that early intervention reduces complications and cost. Suspected COVID-19 cases should be validated by a laboratory test. Currently in Cameroon the nucleic acid amplification test (NAAT): the real time-polymerase chain reaction (RT-PCR) test is being used alongside the recent introduction of the rapid diagnostic test (RDT). Despite the accuracy/specificity of the former, it is quite labour-intensive requiring experts with a long turnaround time from sample collection to test result availability at the point of care [4]. RT-PCR test is done mainly at regional and referral levels through a complex hierarchical procedure that takes up to 2 days or more in some cases for results to be available. The RDT normally should resolve this problem as results should be available within two hours. However, patients are not benefitting fully from RDT as the time from being a suspected case to specimen collection and obtaining results is unnecessarily long. This is due to fragmentation of roles and the lack of autonomy in managing activities related to COVID-19 in clinical settings. Furthermore, the major challenge in this area of diagnostics is the inability to run as many tests as possible for suspected cases in areas with relatively high numbers of COVID-19 cases as well as the inability to effectively conduct contact tracing.

Clinicians and patients suffer the consequences of the aforementioned challenges. While clinicians are obligatorily expected to constantly be protected from becoming infected [5,6], there is increasing fear amongst them of contracting COVID-19 in clinical settings. Clinicians are afraid of being exposed for long periods in taking histories and examining patients who present with symptoms suggestive of COVID-19 and following these suspects in pre-isolation units because of the lack of the necessary personal protective equipment for themselves. The long diagnostic delays of COVID-19 infection equally heighten these fears among clinicians providing care during this period. The consequences of the present situation also affect the majority of patients using clinical facilities during this period in one way or the other. With a growing paranoid attitude among clinicians, patients presenting with episodes of cough and/or fever are easily over suspected as having Covid- 19 without a proper clinical examination. Patients are not well interrogated and examined before a diagnosis is suspected or made given the non-specific aspect of the clinical features of COVID-19. As such patients presenting with other well-known common pulmonary diseases [7,8] are not diagnosed correctly. Development of complications and even fatal outcomes for non COVID-19 cases is on the rise. Over diagnosis of Coivid-19 suspects also leads to some problems. Many are kept for long periods in the pre-isolation unit while awaiting results of the diagnostic tests; a situation which can lead to profound psychological stress for these suspects. Mixing of potentially real COVID-19 cases and non-cases in these units which are generally poorly constructed and overcrowded [6,9] for prolonged periods can also lead to the spread of the disease to non Covid- 19 cases. Delays in establishing COVID-19 diagnosis also slow down interventions to trace potential contacts for isolation and quarantine measures; this can lead to the further spread of the disease. The management of such cases is often sub optimal until a confirmatory test result is available. Some of these conditions in combination with or without other existing co-morbidities may deteriorate very rapidly leading to respiratory failure with possible fatal outcomes. In the area of clinical case management of those with severe COVID-19 disease, a majority of the health facilities lack intensive care units (ICUs) and those that have, do not have adequate equipment and materials to cater for those who present with severe respiratory distress. Last but not the least, rehabilitation professionals are not mobilized to address the rehabilitation needs of the survivors of this disease. This may result in an increase in disabilities among the survivors of the disease.

## Conclusion

It is a well acknowledged fact that providing adequate health care globally during this period of the COVID-19 pandemic especially in developing countries is daunting. Several interrelated factors make it even more complex for the health personnel to arrive at an informed decision timely for optimal care of patients with or without COVID-19. Despite these challenges, clinicians are obliged to provide the best available care without compromise to all patients. It is imperative to acknowledge these challenges and consequences so as to develop strategies to improve on care in clinical settings during this period.
